# Quality of recovery after arthroscopic meniscectomy with remimazolam or propofol anesthesia: A randomized controlled trial

**DOI:** 10.1371/journal.pone.0360759

**Published:** 2026-10-15

**Authors:** Eunah Cho, Ha Yan Kim, Yangjin Kim, Seokyung Shin

**Affiliations:** 1 Department of Anesthesiology and Pain Medicine, Kangbuk Samsung Hospital, Sungkyunkwan University School of Medicine, Seoul, Republic of Korea; 2 Biostatistics Collaboration Unit, Department of Biomedical Systems Informatics, Yonsei University College of Medicine, Seoul, Republic of Korea; 3 Department of Anesthesiology and Pain Medicine, Severance Hospital, Anesthesia and Pain Research Institute, Yonsei University College of Medicine, Seoul, Republic of Korea; Sapienza University of Rome: Universita degli Studi di Roma La Sapienza, ITALY

## Abstract

**Background:**

Anesthetic technique may influence postoperative quality of recovery (QoR). Remimazolam may offer hemodynamic stability and favorable early recovery characteristics, but its effect on patient-reported QoR compared with propofol remains unclear. This is particularly relevant in minor orthopedic surgery, where QoR is a principal outcome. This randomized controlled trial compared postoperative QoR between propofol-based and remimazolam-based total intravenous anesthesia (TIVA) using the QoR-40 questionnaire.

**Materials and methods:**

Eighty adult patients undergoing elective arthroscopic meniscectomy were randomly assigned to propofol-based or remimazolam-based TIVA; 71 patients completed the QoR-40 questionnaire and were included in the final analysis (36 in the Propofol group, 35 in the Remimazolam group). The primary outcome was the global QoR-40 score on postoperative day (POD) 1. Secondary outcomes included intraoperative hemodynamic variables, anesthetic and analgesic requirements, postoperative recovery profiles, pain scores, and postoperative nausea and vomiting.

**Results:**

Global QoR-40 scores on POD 1 did not differ significantly between the Propofol and Remimazolam groups (166.1 ± 16.8 vs. 170.6 ± 13.6; mean difference −4.5, 95% CI −11.8 to 2.7; P = 0.218). In an exploratory analysis, the emotional-state domain scores were higher in the Remimazolam group (37.0 ± 4.3 vs. 39.2 ± 2.5; mean difference −2.2, 95% CI −3.9 to −0.6; P = 0.009). The Remimazolam group had a longer time to full alertness (18 [15–24] vs. 37 [31–45] min; P < 0.001) and longer post-anesthesia care unit stay (30 [30–39] vs. 38 [30–50] min; P = 0.008). Intraoperative hypotension requiring ephedrine occurred more frequently in the Propofol group (44.4% vs. 17.1%; P = 0.013).

**Conclusions:**

Global QoR-40 scores on POD 1 did not differ significantly between propofol-based and remimazolam-based TIVA in adults undergoing elective arthroscopic meniscectomy, although remimazolam was associated with greater intraoperative hemodynamic stability at the cost of slower return to full alertness.

## Introduction

Optimizing postoperative recovery is a central goal of perioperative care. Quality of recovery is closely linked to patient satisfaction and is associated with shorter hospital stay and earlier restoration of normal function [1]. Conventional assessments of postoperative recovery predominantly focused on vital signs, duration of hospital stay, and major clinical outcomes such as morbidity and mortality [2]. However, there is a growing emphasis on evaluating physical and psychological comfort as well as overall quality of life, for a more comprehensive understanding of the patients’ postoperative recovery process [2,3]. Patient-reported outcomes are increasingly recognized as important, as they reflect the patients’ perspective on recovery after surgery [4,5]. A commonly used tool is the Quality of Recovery-40 (QoR-40) questionnaire, which assesses five domains: physical comfort, physical independence, emotional state, psychological support, and pain [2,6]. The QoR-40 has been validated in previous studies across a range of surgeries and anesthesia techniques [1,7,8].

Among the various factors that may impact postoperative quality of recovery (QoR), choice of anesthetic is considered to play a potential role [9]. Propofol and remimazolam are both intravenous (IV) anesthetics with rapid onset and favorable recovery profiles and have been the focus of several comparative studies [10–12]. Emerging evidence indicates that differences in their mechanisms of action, along with variable effects on anesthetic depth, hemodynamic stability, and stress responses, may differentially influence QoR [9,13,14]. Accordingly, several studies have recently compared QoR outcomes between these two anesthetics across diverse surgical settings [9,15–17]. Nevertheless, whereas propofol has been extensively evaluated in relation to other anesthetic agents, remimazolam remains a comparatively new drug, and evidence regarding its impact on postoperative recovery across surgical populations is still limited.

Arthroscopic meniscectomy is among the most frequently performed orthopedic procedures [18], with an emphasis on early mobilization and a rapid return to daily activities [19]. Given that most patients are discharged within one day of surgery, patient-reported QoR during the immediate postoperative period is of particular relevance. We aimed to compare QoR between general anesthesia with propofol and remimazolam in patients undergoing arthroscopic meniscectomy by utilizing the QoR-40 questionnaire. We hypothesized that differences between the two anesthetics would result in different postoperative recovery profiles.

## Materials and methods

### Ethics

The protocol of this study (protocol number 4-2021-1803) was approved by the Institutional Review Board of Severance Hospital, Yonsei University Health System, Seoul, Republic of Korea (Chairperson Prof Jin Seok Kim) on February 2, 2022 and registered at http://clinicaltrials.gov (NCT05280236). Written informed consent was obtained from all study participants prior to enrollment. This study was conducted from March 14, 2022, to April 30, 2024 at a single tertiary hospital (Severance Hospital, Seoul, Republic of Korea) in compliance with the principles of the Declaration of Helsinki.

### Study participants

Adult patients aged 19 or older and American Society of Anesthesiologists (ASA) Physical Status I - III scheduled for elective arthroscopic meniscectomy under general anesthesia were consecutively screened and enrolled. Patients with any of the following criteria were excluded from the study: refusal to participate, airway-related diseases such as upper respiratory tract infection, uncontrolled asthma, or pneumonia, known allergies to propofol or benzodiazepines, impaired renal or hepatic function, cardiac dysfunction with left ventricular ejection fraction < 55%, pregnancy or breastfeeding, and history of substance abuse.

### Randomization and blinding

Patients were randomly assigned to either the Propofol group or the Remimazolam group on the day before surgery in a 1:1 allocation ratio. The random allocation sequence was generated using a computer-generated random permuted block randomization algorithm by an anesthesiologist who was not involved in patient enrollment, anesthesia management, or outcome assessment, and allocations were sealed in sequentially numbered opaque envelopes. Each envelope was opened by the attending anesthesiologist only immediately before induction, after enrollment was complete, so that investigators enrolling patients had no advance access to the allocation sequence. Due to the apparent difference in the infusion methods of propofol and remimazolam, the attending anesthesiologists could not be blinded to group randomization. However, the investigator conducting the surveys and assessments remained blinded to the group assignment, and patients were not aware of the anesthetic they received until all assessments were completed. Syringes containing the study drug were covered with opaque material in the anesthetic preparation room.

### Study protocol

Upon arrival at the operating theater, standard monitoring which included electrocardiography, non-invasive blood pressure, pulse oximetry, and electroencephalography (SEDline® Masimo; Irvine, CA) monitors were applied.

In the Propofol group, anesthesia induction and maintenance were done with propofol (Fresofol MCT 2% 50 ml Inj; Fresenius Kabi Austria GmbH, Graz, Austria) and remifentanil (Ultian; Hanlim Pharm Co. Ltd., Seoul, Republic of Korea) using a target-controlled infusion (TCI) pump (Agilia® SP TIVA, Fresenius Kabi, Bad Homburg, Germany). Propofol and remifentanil TCI was done according to the Marsh model [20] and Minto model [21], respectively. Propofol was initiated at a target effect-site concentration (Ce) of 3.0–5.0 µg·mL^-1^ and remifentanil at a Ce of 3.0 ng·mL^-1^. After loss of consciousness (LOC) was confirmed, a supraglottic airway (SGA) was inserted without neuromuscular blocking agents. During maintenance, propofol Ce was titrated within 2.5–4.0 µg·mL^−1^ to maintain a patient state index (PSI) of 25–50, and remifentanil Ce within 2.0–5.0 ng·mL^−1^.

In the Remimazolam group, 50 mg of remimazolam (Byfavo®; Hana Pharm Co. Ltd., Seoul, Republic of Korea) was diluted with normal saline and prepared at a concentration of 1 mg·mL^-1^ and loaded into a general-purpose syringe pump (TE-332 pump; Terumo, Tokyo, Japan). Remifentanil was prepared in the same manner as the Propofol group. Anesthesia induction was performed by administering remimazolam at 12.0 mg·kg^-1^·h^-1^ with remifentanil TCI at Ce 3.0 ng·mL^-1^ until LOC was confirmed, followed by SGA insertion. Anesthesia was maintained with remimazolam infusion at 0.8–2.0 mg·kg^-1^·h^-1^, titrated to maintain PSI between 25 and 50. The maximum remimazolam infusion rate was capped at 2.0 mg·kg^-1^·h^-1^. Remifentanil TCI was titrated within 2.0–5.0 ng·mL^-1^, as in the Propofol group.

Mechanical ventilation was standardized (tidal volume 6–8 mL·kg^−1^) and adjusted to maintain end-tidal CO_2_ (ETCO_2_) at 35–40 mmHg. Intraoperative hypotension (systolic blood pressure <90 mmHg or mean blood pressure <60 mmHg) was treated with ephedrine 4–8 mg as needed. IV acetaminophen 1 g infusion was started at anesthesia induction, and fentanyl 50 µg with ramosetron 0.3 mg were given at the end of surgery. After confirming adequate self-respiration with sufficient tidal volume, the SGA was removed, and the patients were transferred to the post-anesthesia care unit (PACU).

Postoperative pain was assessed using an 11-point verbal numerical rating scale (VNRS), where 0 indicates no pain and 10 indicates the worst pain imaginable. In the PACU, patients with VNRS ≥ 4 were given IV fentanyl as rescue analgesia, with the dose determined by the attending anesthesiologist according to patient age and weight. All patients were scheduled to be discharged on POD 1. In accordance with the orthopedic department’s postoperative protocol, IV acetaminophen 1 g was administered once on the afternoon of surgery and once on the morning of POD 1. Ward rescue analgesia was available for a VNRS score ≥ 4 and consisted of IV tramadol 25–50 mg or IV pethidine 25–50 mg, with the choice of agent and dose determined by the attending surgeon. Postoperative nausea and vomiting (PONV) were assessed using the following scale: 0 = no symptoms; 1 = mild nausea; 2 = severe nausea requiring antiemetic treatment; 3 = retching or vomiting. A score of 2 or higher was considered as significant PONV, and metoclopramide 10 mg IV was administered.

### Primary outcome: QoR-40 at postoperative day 1

The QoR-40 questionnaire has been previously validated across different languages and patient populations [1,2], and consists of 40 items across five dimensions: emotional state (9 items), physical comfort (12 items), psychological support (7 items), physical independence (5 items), and pain (7 items). Each item is rated on a 5-point Likert scale (none, sometimes, moderate, mostly, always). The sum of all item scores is calculated to obtain the global QoR-40 score, and can range from 40 (worst quality of recovery) to 200 (best quality of recovery) [2]. Patient QoR was assessed at two time points: on the evening before surgery as baseline and approximately 24 hours after surgery before discharge, corresponding to postoperative day (POD) 1.

### Secondary outcomes

Mean arterial pressure (MAP) and heart rate (HR) were recorded at predefined time points: before and immediately after induction of anesthesia, at surgical incision, at the end of surgery, immediately after SGA removal, upon PACU admission, and at PACU discharge. PSI levels were documented at the same time points except for PACU admission and discharge.

The total dose and frequency of ephedrine administration during anesthesia were recorded, together with sedative and remifentanil consumption. Drug consumption was expressed as two weight-adjusted infusion rates: the overall infusion rate, calculated over the entire anesthesia period from induction to the end of anesthesia, and the maintenance infusion rate, calculated from surgical incision to the end of surgery, thereby excluding the induction and emergence periods. Duration of anesthesia, surgery, and PACU stay were also documented, with surgical duration defined as the interval from the first surgical incision to final wound closure. Outcomes related to recovery included the time from discontinuation of the study drug to SGA removal, first verbal communication, and full alertness. Full alertness was defined as the patient being awake and remaining so without stimulation, and able to correctly state person, place, and time and communicate appropriately. Alertness was assessed every 5 minutes from PACU admission by the investigator who was blinded to group assignment.

Postoperative outcomes included the highest pain score at the PACU, pain score at discharge from the PACU, on POD 0, and POD 1, as well as whether rescue analgesia was required. PONV scores were assessed and recorded at the PACU and on POD 0 and POD 1.

### Statistical analysis

The primary endpoint was the global QoR-40 score on POD 1. In preliminary data from six consecutive patients assessed by the authors (unpublished data), the mean POD 1 global QoR-40 score was 171 (SD 9.20). Based on a minimal clinically important difference (MCID) of 6.3 points for the QoR-40 [22], 35 participants per group were required for a two-sided independent t-test with an alpha of 5% and 80% power. Allowing for a 15% dropout rate, 40 participants were enrolled per group (total 80), as registered. The 71 patients included in the primary analysis exceeded the required total of 70.

Normality was assessed using the Shapiro–Wilk test. Continuous variables are expressed as mean ± SD or median (interquartile range, IQR) according to distribution, and categorical variables as numbers (%). Between-group comparisons were performed using Student’s t-test for normally distributed continuous variables, the Mann–Whitney U test for non-normally distributed variables, and the chi-square test or Fisher’s exact test for categorical variables.

The primary analysis included all randomized patients who received the allocated intervention and had a non-missing POD 1 global QoR-40 score (complete-case analysis). The primary treatment effect was defined as the between-group difference in mean POD 1 global QoR-40 score (propofol minus remimazolam), estimated with a 95% confidence interval (CI) and tested using an independent t-test. Because the global QoR-40 score was not normally distributed, a supporting non-parametric analysis was performed using the Mann–Whitney U test, with the Hodges–Lehmann location shift and its 95% CI. Baseline balance was assessed using absolute standardized mean differences (SMDs), with an SMD > 0.20 indicating meaningful imbalance. To test robustness to chance imbalance, the primary outcome was reanalyzed in a linear model including treatment group and the imbalanced baseline characteristics as covariates; where two imbalanced variables were collinear measures of the same construct, only one was retained.

Missing POD 1 data were addressed by multiple imputation using fully conditional specification with 20 imputations (random seed 12345), including all 80 randomized patients; the imputation model included treatment group, age, body mass index, ASA physical status, asthma, and the preoperative QoR-40 domain scores, and estimates were pooled using Rubin’s rules. Because multiple imputation assumes data are missing at random, characteristics of patients with and without questionnaire data were compared (S1 Table).

Secondary and domain-specific outcomes — QoR-40 domains, hemodynamic variables, anesthetic and vasoactive requirements, recovery profiles, pain scores, rescue analgesia, and PONV — were analyzed without multiplicity adjustment and are exploratory.

Perioperative repeated measures (MAP, HR, PSI) were analyzed using linear mixed-effects models with fixed effects for treatment group, categorical time, and their interaction. No random effects were included; repeated measurements within each participant were modeled using an unstructured covariance matrix, and the pre-induction measurement was included as the first level of the repeated-measures time factor rather than being entered as a separate covariate. MAP and HR were modeled across five intraoperative and two post-anesthesia care unit time points, and PSI across five intraoperative time points. Fixed effects were evaluated using Type 3 tests, and between-group comparisons at individual time points were interpreted as exploratory without adjustment for multiplicity. Statistical significance was defined as P < 0.05. All analyses were conducted using SAS 9.4 (SAS Institute Inc., Cary, NC, USA).

## Results

The flow diagram of the study is shown in Fig 1. Eighty patients were enrolled and randomized to the Propofol group (n = 40) or the Remimazolam group (n = 40). All 80 patients received the allocated intervention and completed the study procedures. Postoperative QoR-40 questionnaires were not administered to 9 patients (4 in the Propofol group and 5 in the Remimazolam group) due to administrative failure to distribute the questionnaire, rather than patient refusal, withdrawal, or inability to complete it; all 9 patients had complete preoperative QoR-40 scores and postoperative clinical data. The proportion of patients without postoperative QoR-40 data did not differ between groups (10.0% vs. 12.5%; P > 0.999). Accordingly, 71 patients were included in the primary complete-case analysis, with 36 in the Propofol group and 35 in the Remimazolam group. A comparison of patients with and without postoperative QoR-40 data is presented in S1 Table.

**Fig 1:**
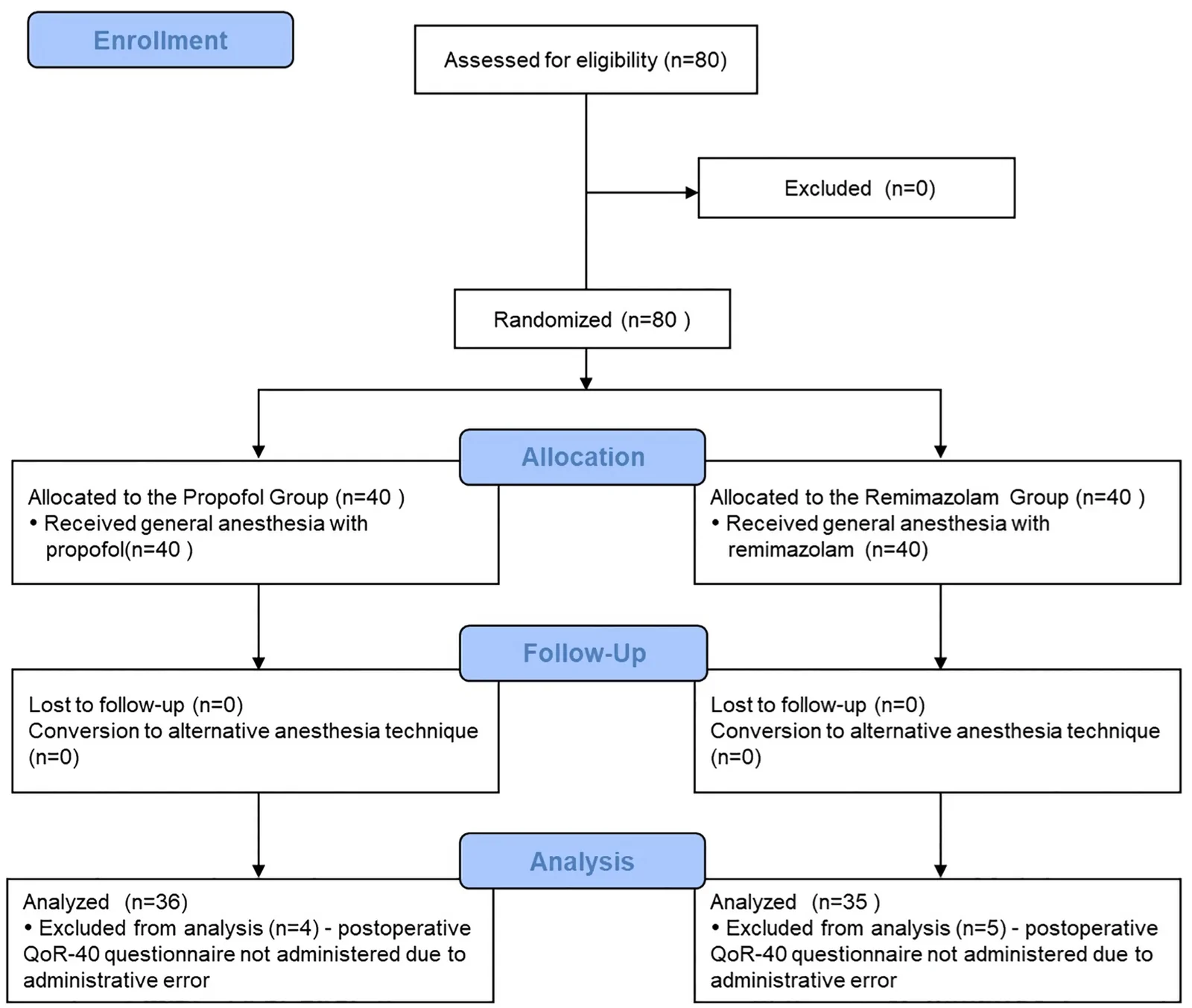
CONSORT diagram of patient recruitment.

No baseline characteristic differed significantly between the two groups (Table 1). Age, weight, BMI, ASA physical status, and asthma exceeded the prespecified imbalance threshold (standardized mean difference > 0.20); weight was excluded from adjusted models owing to collinearity with BMI. The QoR-40 scores are summarized in Table 2. The preoperative QoR-40 scores were not different between the two groups (P = 0.872). On POD 1, the global QoR-40 score was 166.1 ± 16.8 in the Propofol group and 170.6 ± 13.6 in the Remimazolam group, with a mean difference of −4.5 points (95% CI, −11.8 to 2.7; P = 0.218). The Hodges-Lehmann location shift in the supporting non-parametric analysis was −3.0 points (95% CI, −12.0 to 3.0; P = 0.306; S3 Table). The adjusted mean difference was −5.0 points (95% CI, −11.1 to 1.0; P = 0.100) in the baseline-adjusted sensitivity analysis and −5.4 points (95% CI, −11.6 to 0.8; P = 0.088) in the multiple imputation analysis including all 80 randomized patients (Table 3). In exploratory domain-specific analyses, the POD 1 emotional state domain score was higher in the Remimazolam group (mean difference −2.2 points; 95% CI, −3.9 to −0.6; P = 0.009); scores for other dimensions did not differ. A relatively greater decrease in global scores on POD 1 was observed in the Propofol group, but the difference was not significant (19.9 ± 14.5 vs. 14.0 ± 10.5; P = 0.054). Domain-level sensitivity analyses are presented in S2 Table.

**Table 1:** Baseline characteristics of the study population.

	Propofol group	Remimazolam group	SMD	P−value
	n = 36	n = 35		
Age, years	52.2 ± 13.0	48.7 ± 14.8	0.248	0.300
Sex, male, n (%)	16 (44.4)	18 (51.4)	−0.140	0.556
Height, cm	166.7 ± 9.6	167.8 ± 10.8	−0.114	0.633
Weight, kg	67.8 ± 11.3	74.0 ± 20.0	−0.380	0.116
Body mass index, kg·m^-2^	24.3 ± 2.8	25.9 ± 4.7	−0.416	0.086
ASA physical class, n (%)			0.389	0.287
I	20 (55.6)	19 (54.3)		
II	10 (27.8)	14 (40.0)		
III	6 (16.7)	2 (5.7)		
Hypertension, n (%)	8 (22.2)	8 (22.9)	−0.015	0.949
Diabetes mellitus, n (%)	2 (5.6)	3 (8.6)	−0.118	0.674
Asthma, n (%)	3 (8.3)	1 (2.9)	0.240	0.614

Data are presented as mean ± standard deviation (SD), or number (%).

An absolute SMD > 0.20 was considered to indicate meaningful imbalance.

ASA, American Society of Anesthesiologists, SMD, standardized mean difference.

**Table 2:** Quality of Recovery−40 questionnaire scores.

	Propofol group	Remimazolam group	Mean difference (95% CI)	P−value
	n = 36	n = 35		
Preoperative baseline				
Emotional state	40.5 ± 4.8	40.6 ± 3.5	0.0 (−2.1 to 2.0)	0.966
Physical comfort	57.6 ± 2.5	56.9 ± 3.5	0.7 (−0.7 to 2.1)	0.339
Psychological support	32.1 ± 4.0	31.7 ± 4.9	0.4 (−1.7 to 2.5)	0.708
Physical independence	23.8 ± 2.1	23.5 ± 2.8	0.3 (−0.9 to 1.4)	0.615
Pain	32.1 ± 2.8	32.1 ± 3.2	0.0 (−1.4 to 1.4)	0.972
Global score	186.0 ± 11.5	184.6 ± 14.2	1.4 (−4.7 to 7.5)	0.656
Postoperative day 1				
Emotional state	37.0 ± 4.3	39.2 ± 2.5	−2.2 (−3.9 to −0.6)	0.009*
Physical comfort	51.7 ± 5.9	53.5 ± 3.6	−1.8 (−4.1 to 0.6)	0.135
Psychological support	30.8 ± 4.1	30.2 ± 5.2	0.6 (−1.6 to 2.8)	0.585
Physical independence	17.8 ± 4.0	18.3 ± 3.9	−0.5 (−2.4 to 1.4)	0.611
Pain	28.8 ± 4.1	29.5 ± 3.3	−0.7 (−2.4 to 1.1)	0.464
Global score	166.1 ± 16.8	170.6 ± 13.6	−4.5 (−11.8 to 2.7)	0.218
Preoperative−postoperative global score difference	19.9 ± 14.5	14.0 ± 10.5	5.9 (−0.1 to 11.9)	0.054

Data are presented as mean ± standard deviation.

Differences are mean differences with 95% confidence intervals, calculated as Propofol group minus Remimazolam group, and P values are from the independent t−test. A supporting non−parametric analysis is presented in S3 Table.

CI, confidence interval

*P < 0.05

**Table 3:** Sensitivity analyses of the global QoR − 40 score on postoperative day 1.

Analysis	n	Adjusted mean difference (95% CI)	P−value
Complete−case analysis	71	−5.0 (−11.1 to 1.0)	0.100
Multiple imputation analysis	80	−5.4 (−11.6 to 0.8)	0.088

Differences are calculated as Propofol group minus Remimazolam group

Both models were adjusted for age, ASA physical status, asthma, body mass index (BMI), and preoperative global QoR − 40 score. Domain−level results are presented in S2 Table.

CI, confidence interval; QoR − 40, Quality of Recovery−40; ASA, American Society of Anesthesiologists.

*P < 0.05

Exploratory intraoperative outcomes and recovery profiles are summarized in Table 4. A significantly greater proportion of the patients of the Propofol group required ephedrine to treat hypotension compared to the Remimazolam group (44.4% vs. 17.1%, P = 0.013), and ephedrine dose requirements were also higher (P = 0.017). Patients of the Remimazolam group [0.20 (0.17–0.25) µg·kg^−1^·min^−1^] required a significantly higher infusion rate of remifentanil compared to the Propofol group [0.18 (0.14–0.20) µg·kg^−1^·min^−1^] during surgery (P = 0.014). The time from the discontinuation of the anesthetic agent to SGA removal was shorter in the Remimazolam group compared to the Propofol group (10.7 ± 2.9 min vs. 12.9 ± 3.8 min, P = 0.005). The time to first communication did not differ between the groups (P = 0.124), whereas the time to full alertness was shorter in the Propofol group than in the Remimazolam group [18.0 (15.0–24.0) min vs. 37.0 (31.0–45.0) min; P < 0.001]. The duration of PACU stay was also shorter in the Propofol group [30.0 (30.0–39.0) min vs. 38.0 (30.0–50.0) min, P = 0.008]. No patient in either group received a neuromuscular blocking agent.

**Table 4:** Exploratory intraoperative data and recovery profiles.

	Propofol group	Remimazolam group	Difference (95% CI)	P−value
	n = 36	n = 35		
Ephedrine requirement				
Number of patients, n (%)	16 (44.4)	6 (17.1)	27.3 (6.8 to 47.8)	0.013*
Dose, mg†	0.0 (0.0–6.0)	0.0 (0.0–0.0)	0.0 (0.0 to 4.0)	0.017*
Anesthesia duration, min†	60.0 (55.0–70.0)	60.0 (55.0–75.0)	0.0 (−5.0 to 5.0)	0.857
Surgery duration, min†	30.0 (29.5–36.0)	30.0 (23.0–41.0)	3.0 (0.0 to 7.0)	0.144
Propofol consumption, mg·kg ^− 1^·h ^− 1^				
Overall infusion rate	7.7 ± 1.5	–	–	–
Maintenance infusion rate	8.1 ± 1.7	–	–	–
Remimazolam consumption, mg·kg ^− 1^·h ^− 1^				
Overall infusion rate	–	1.3 ± 0.3	–	–
Maintenance infusion rate	–	1.4 ± 0.4	–	–
Remifentanil consumption, µg·kg ^− 1^·min ^− 1^				
Overall infusion rate	0.09 ± 0.02	0.10 ± 0.03	−0.01 (−0.02 to 0.01)	0.269
Maintenance infusion rate†	0.18 (0.14–0.20)	0.20 (0.17–0.25)	−0.03 (−0.05 to −0.004)	0.014*
Time to SGA removal, min	12.9 ± 3.8	10.7 ± 2.9	2.3 (0.7 to 3.9)	0.005*
Time to first communication, min†	13.0 (11.0–16.0)	14.0 (13.0–16.0)	−1.0 (−3.0 to 0.0)	0.124
Time to fully alert status, min†	18.0 (15.0–24.0)	37.0 (31.0–45.0)	−17.0 (−21.0 to −13.0)	<0.001*
PACU duration, min†	30.0 (30.0–39.0)	38.0 (30.0–50.0)	−5.0 (−10.0 to 0.0)	0.008*

Data are presented as mean ± standard deviation (SD), median (interquartile range), or number (%). † denotes non−normally distributed variables.

Differences are presented with 95% confidence intervals as Propofol group minus Remimazolam group. Mean differences, Hodges–Lehmann estimates, and risk differences were used as appropriate.

SGA, supraglottic airway; PACU, post anesthesia care unit; CI, confidence interval.

* P < 0.05

Perioperative vital signs are shown in Fig 2. Intraoperative MAP was higher in the Remimazolam group at surgical incision (P = 0.050), and HR was consistently lower in the Propofol group, with significant differences after induction, at surgical incision, at end of surgery, and at both PACU time points (S4 Table). PSI values were lower in the Propofol group at all intraoperative time points after induction (all P < 0.001), and this pattern was unchanged after adjustment for imbalanced baseline characteristics (Table 5).

**Table 5:** Perioperative patient state index (PSI).

	Unadjusted			Baseline−adjusted		
Time point	Propofol group n = 36	Remimazolam group n = 35	P−value	Propofol group n = 36	Remimazolam group n = 35	P−value
Before induction	93.2 ± 0.4	92.5 ± 0.4	0.172	94.4 ± 0.6	93.9 ± 0.6	0.289
After induction	37.7 ± 1.8	47.4 ± 1.8	<0.001*	38.9 ± 1.8	48.8 ± 1.9	<0.001*
Surgical incision	28.9 ± 1.6	43.5 ± 1.6	<0.001*	30.2 ± 1.7	45.0 ± 1.7	<0.001*
End of surgery	28.8 ± 1.5	38.7 ± 1.5	<0.001*	30.1 ± 1.6	40.1 ± 1.6	<0.001*
SGA removal	62.7 ± 2.3	68.2 ± 2.4	0.104	64.0 ± 2.4	69.6 ± 2.4	0.095

Data are presented as estimated marginal mean ± standard error from linear mixed−effects models.

The baseline−adjusted model was additionally adjusted for the baseline characteristics that were imbalanced between groups (standardized mean difference > 0.20): age, ASA physical status, asthma, and body mass index.

PSI, patient state index; SGA, supraglottic airway; ASA, American Society of Anesthesiologists.

*P < 0.05

**Fig 2:**
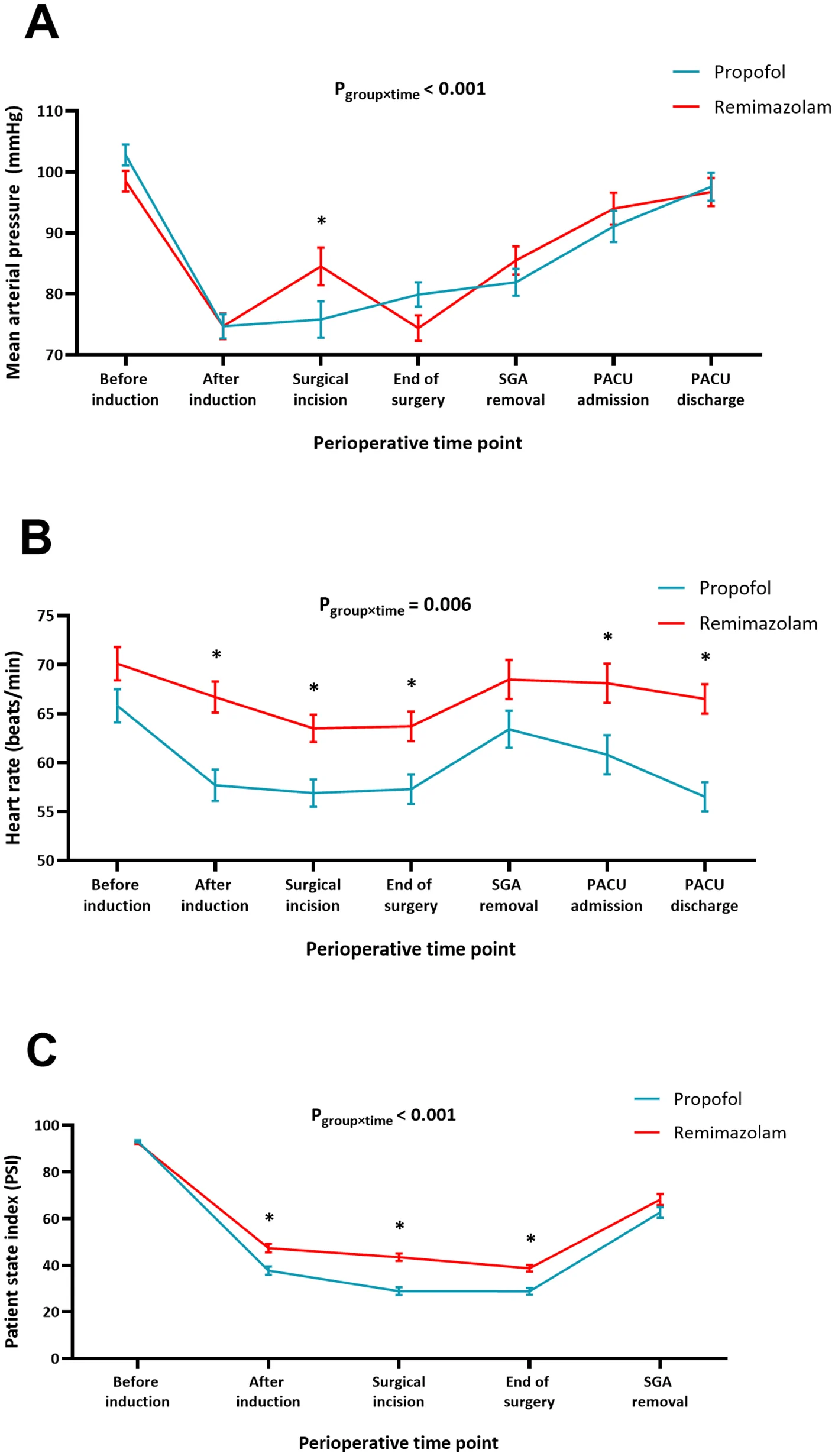
Graphs describing mean arterial pressure (A), heart rate (B), and patient state index (C) measured during the study period between the Propofol group (blue) and the Remimazolam group (red). Mean arterial pressure differed between groups over time (P_group×time_ < 0.001). Heart rate was lower in the Propofol group over time (P_group×time_ = 0.006). Patient state index was higher in the Remimazolam group over time (P_group×time_ < 0.001). Asterisks indicate exploratory between-group comparisons at individual time points.

In exploratory analyses, pain scores at the PACU, on POD 0, and on POD 1 did not differ between the two groups. Rescue analgesia in the PACU was required by 6 of 36 patients (16.7%) in the Propofol group and 1 of 35 (2.9%) in the Remimazolam group (P = 0.107). All rescue analgesia administered in the PACU was intravenous fentanyl, at doses of 50 µg in five patients and 100 µg in one patient in the Propofol group, and 25 µg in one patient in the Remimazolam group. PONV scores did not differ between groups at any time point, and no patient experienced significant PONV in the PACU (Table 6). All patients were discharged on POD 1 as planned and no serious adverse events were recorded in either group.

**Table 6:** Exploratory postoperative pain, use of rescue analgesics, and postoperative nausea and vomiting.

	Propofol group	Remimazolam group	Difference (95% CI)	P−value
	n = 36	n = 35		
Pain				
Highest score at PACU	2 (2–3)	2 (1–2)	0.0 (0.0 to 1.0)	0.090
At discharge from PACU	2 (1–2)	2 (1-2)	0.0 (0.0 to 1.0)	0.213
POD 0	3 (2–7)	2 (2-7)	0.0 (0.0 to 1.0)	0.588
POD 1	2 (2–2.5)	2 (2−2)	0.0 (0.0 to 0.0)	0.841
Patients requiring rescue analgesia, n (%)				
PACU	6 (16.7)	1 (2.9)	13.8 (0.4 to 27.2)	0.107
POD 0	19 (52.8)	15 (44.1)	8.7 (−14.7 to 32.0)	0.485
POD 1	13 (36.1)	7 (20.0)	16.1 (−4.4 to 36.6)	0.188
PONV, NRS 0–3				
PACU	0 (0−0)	0 (0−0)	–	–
POD 0	0 (0–1)	0 (0–1)	0.0 (0.0 to 0.0)	0.923
POD 1	0 (0−0)	0 (0−0)	0.0 (0.0 to 0.0)	0.960
Significant PONV, (NRS ≥ 2), n (%)				
PACU	0 (0.0)	0 (0.0)	–	–
POD 0	4 (11.1)	4 (11.4)	−0.3 (−15.0 to 14.4)	>0.999
POD 1	3 (8.3)	1 (2.9)	5.5 (−5.1 to 16.1)	0.614

Data are presented as median (interquartile range) or number (%).

Differences are presented with 95% confidence intervals as Propofol group minus Remimazolam group. Hodges–Lehmann estimates and risk differences were used as appropriate. Risk differences are presented with Wald 95% confidence intervals, whereas P values for binary outcomes are from Fisher’s exact test. Because these are calculated by different methods, a confidence interval may exclude zero while the corresponding test is not significant.

All rescue analgesia administered in the PACU was intravenous fentanyl: 50 µg in five patients and 100 µg in one patient in the Propofol group, and 25 µg in one patient in the Remimazolam group.

PACU, post anesthesia care unit; POD, postoperative day; PONV, postoperative nausea and vomiting; NRS, numeric rating scale; CI, confidence interval.

* P < 0.05

## Discussion

Historically, general anesthesia has mostly been performed by either propofol-based total intravenous anesthesia (TIVA) or volatile agent-based anesthesia. However, the recently developed remimazolam has added to the armamentarium of agents that can be used to induce and maintain general anesthesia. While propofol-based TIVA is considered to improve QoR by reducing postoperative nausea and vomiting and emergence delirium compared to volatile agent-based anesthesia [23,24], the available evidence has not identified a statistically significant difference in QoR after general anesthesia between propofol and remimazolam-based TIVA [25]. In the present study, we did not detect a statistically significant difference in global QoR-40 score on POD 1 between the two groups. This should not be taken as evidence that the two techniques produce equivalent recovery quality; the confidence interval (−11.8 to 2.7 points) extends beyond the MCID of 6.3 points, so a real advantage for remimazolam remains possible. While all participants in our study were observed overnight as inpatients per hospital protocol, arthroscopic meniscectomies are often performed as outpatient procedures, and the observed differences in postoperative QoR-40 scores may be more relevant in such settings where rapid patient discharge is typical.

Although propofol and remimazolam both act on γ-aminobutyric acid subtype A (GABA_A_) receptors, they have distinct clinical profiles [9,14,26,27]. Consistent with previous reports of greater hemodynamic stability with remimazolam [12,16], fewer patients in the Remimazolam group required ephedrine for hypotension (17.1% vs 44.4%; P = 0.013), and mean arterial pressure was higher at surgical incision. Whether this translated into better organ perfusion [28] or reduced postoperative fatigue cannot be determined from our data, as perfusion-related endpoints were not measured.

This hemodynamic difference occurred despite higher remifentanil consumption in the Remimazolam group. The greater remifentanil requirement during maintenance may reflect differences in the overall hypnotic–analgesic interaction between the two anesthetic regimens, although the underlying pharmacodynamic mechanism remains uncertain. A preclinical primate study showed that the sedative synergy between remimazolam and remifentanil was markedly greater than that between propofol and remifentanil. This synergy, however, relates to the level of sedation rather than to analgesia, and therefore does not directly account for the difference in opioid requirement [29]. One possible explanation is that propofol may provide a greater degree of muscle relaxation compared to remimazolam, as suggested by studies comparing propofol to other IV anesthetics [30]. This may be particularly relevant because neuromuscular blocking agents were not used in this study. However, because muscle tone, patient movement, and surgical conditions were not objectively assessed, whether these factors contributed to the higher remifentanil requirement in the Remimazolam group could not be determined. Despite the higher intraoperative remifentanil infusion, fewer patients in the Remimazolam group required rescue analgesia in the PACU, although this difference did not reach statistical significance. Similar findings have been reported previously [16,17]. With only seven patients requiring rescue analgesia in total, however, our data cannot distinguish between a possible anti-hyperalgesic effect of remimazolam and deeper residual sedation during early recovery.

In a previous study, Tang et al. compared QoR-15 scores, which is a simplified version of the QoR-40, in patients undergoing arthroscopic meniscus repair and found scores to be higher with remimazolam compared to propofol [9]. However, this difference was smaller than the MCID for the QoR-15. This study observed longer sleep durations and better sleep quality after anesthesia with remimazolam and suggested this may have contributed to higher scores in dimensions of physical independence and psychological support. Although we were not able to observe differences in either of these domains, our exploratory analysis found that scores were higher with remimazolam in the domain of emotional state (mean difference −2.2 points, 95% CI −3.9 to −0.6). This difference persisted after adjustment for imbalanced baseline characteristics and in the multiple imputation analysis, and was also evident in the non-parametric analysis. Nonetheless, the domain analyses were not adjusted for multiple comparisons, and no MCID has been established for individual QoR-40 domains, so the clinical relevance of a difference of this magnitude is uncertain. Although remimazolam is a benzodiazepine with anxiolytic and amnesic properties, it remains unclear whether this difference can be attributed to these pharmacologic effects, particularly considering its short context-sensitive half-life [31]. Remimazolam is also well known to cause markedly less injection pain than propofol [32], which may improve the induction experience and contribute to patient satisfaction [33]. However, the incidence of injection pain was not assessed in our study, and whether this would be sufficient to translate into improved QoR scores is unclear.

Despite targeting the same PSI range (25–50), intraoperative PSI values were consistently higher in the Remimazolam group. Because PSI was used to guide anesthetic titration, this finding should be interpreted cautiously and not as an independent clinical outcome. We have previously observed relatively high PSI values with remimazolam [34], but it remains unclear whether this reflects a true difference in anesthetic depth, drug-specific electroencephalographic patterns, or a limitation of the monitoring technology [35]. Although the infusion rates of sedatives and analgesics were within typical ranges, using a symmetrical target for both propofol and remimazolam may have confounded drug dosing, opioid requirements and emergence times. Further studies are needed to determine the reliability of processed electroencephalographic indices for assessing anesthetic depth with remimazolam.

The two anesthetics demonstrated distinct recovery profiles: time to SGA removal was shorter in the Remimazolam group, but the time required to reach a fully alert mental status was markedly prolonged compared to the Propofol group. Earlier SGA removal may have been related to less respiratory depression with remimazolam [36]. In contrast, the delayed return to full alertness may reflect residual benzodiazepine sedative effects, greater hypnotic synergy with remimazolam and remifentanil, as described above, or potentially deeper or prolonged sedation resulting from titration to the predefined PSI target. Notably, the time to first communication did not differ between groups. Because full alertness additionally required sustained wakefulness without stimulation, this suggests the delay in the remimazolam group reflected residual sedation rather than a slower return of arousability. Delayed full alertness after remimazolam has also been reported previously [9,37]. All patients were observed in the PACU until they achieved full alertness, contributing to a significantly longer PACU stay for the Remimazolam group. Without the use of routine flumazenil reversal, full recovery after remimazolam anesthesia appears to be slower than with propofol [38].

Our study has several limitations. First, the sample size calculation relied on a preliminary standard deviation estimate derived from only six patients. The standard deviations observed in the final cohort were considerably larger (16.8 and 13.6 in the Propofol and Remimazolam groups, respectively; pooled 15.3). In a sensitivity analysis of statistical power under alternative variance assumptions, power to detect the 6.3-point MCID fell to approximately 64% and 46% in the enrolled sample of 80, and to 59% and 42% in the analyzed sample of 71, assuming standard deviations of 12 and 15 respectively — the latter approximating the observed pooled value. At the observed variability, the study had 80% power only to detect a difference of approximately 10 points. A type II error therefore cannot be excluded. Second, POD 1 QoR-40 data were missing for nine patients (11%) because of administrative failure to distribute the questionnaire rather than patient refusal or inability to complete it. Non-completion was balanced between groups, and multiple imputation including all 80 randomized patients yielded results consistent with the complete-case analysis. Non-completers nonetheless differed from completers in ASA physical status distribution, so residual bias from informative missingness cannot be entirely excluded. Third, although patients and outcome assessors were blinded, the attending anesthesiologists could not be blinded to group allocation because of the different appearance and delivery of the two agents. This may have introduced performance bias and influenced intraoperative management, including remifentanil titration and vasopressor administration. Fourth, our study assessed outcomes only up to postoperative 24 hours. While it is desirable to observe a longer trajectory of recovery, we focused on the immediate postoperative period considering the nature of arthroscopic meniscectomy. A study that includes a longer follow-up period with data on functional outcome would clarify the longer-term effects of anesthetics on outcome. Lastly, flumazenil was not routinely administered, as we intended to characterize the natural recovery profile of remimazolam compared with propofol. Our findings regarding delayed recovery may therefore not be generalizable to settings where routine reversal is used; recovery times, PACU duration, and potentially patient-reported recovery may differ when flumazenil is administered. Future studies are needed to evaluate how flumazenil reversal affects both early recovery profiles and postoperative QoR after remimazolam anesthesia.

In conclusion, global QoR-40 scores on POD 1 did not differ significantly between propofol- and remimazolam-based anesthesia for arthroscopic meniscectomy. Remimazolam was associated with greater intraoperative hemodynamic stability at the cost of a slower return to full alertness, and exploratory analyses suggested a difference in the emotional state domain. These secondary findings were not adjusted for multiple comparisons and should be interpreted cautiously.

## Supporting information

S1 Data De-identified dataset underlying all reported analyses. Includes all 80 randomized patients, with a variable dictionary provided on the second worksheet.
(XLSX)

S1 Table Baseline and perioperative characteristics of completers and non-completers. Comparison of patients with and without a postoperative day 1 QoR-40 score.
(DOCX)

S2 Table Baseline-adjusted and multiple imputation sensitivity analyses of QoR-40 scores on postoperative day 1. (DOCX)

S3 Table Non-parametric analysis of QoR-40 questionnaire scores. Median values with Hodges–Lehmann location shifts.
(DOCX)

S4 Table Perioperative mean arterial pressure and heart rate. Unadjusted and baseline-adjusted estimates from linear mixed-effects models.
(DOCX)

## References

[1] Gornall BF, Myles PS, Smith CL, Burke JA, Leslie K, Pereira MJ, et al. Measurement of quality of recovery using the QoR-40: a quantitative systematic review. Br J Anaesth. 2013;111(2):161–9. doi: 10.1093/bja/aet014 23471753

[2] Myles PS, Weitkamp B, Jones K, Melick J, Hensen S. Validity and reliability of a postoperative quality of recovery score: the QoR-40. Br J Anaesth. 2000;84(1):11–5. doi: 10.1093/oxfordjournals.bja.a013366 10740540

[3] Stark PA, Myles PS, Burke JA. Development and psychometric evaluation of a postoperative quality of recovery score: the QoR-15. Anesthesiology. 2013;118(6):1332–40. doi: 10.1097/ALN.0b013e318289b84b 23411725

[4] Warnakulasuriya SR, Patel RC, Singleton GF, Moonesinghe SR. Patient-reported outcomes for ambulatory surgery. Curr Opin Anaesthesiol. 2020;33(6):768–73. doi: 10.1097/ACO.0000000000000921 33002956

[5] Khullar OV, Fernandez FG. Patient-Reported Outcomes in Thoracic Surgery. Thorac Surg Clin. 2017;27(3):279–90. doi: 10.1016/j.thorsurg.2017.03.007 28647074

[6] Lee JH, Kim D, Seo D, Son J-S, Kim D-C. Validity and reliability of the Korean version of the Quality of Recovery-40 questionnaire. Korean J Anesthesiol. 2018;71(6):467–75. doi: 10.4097/kja.d.18.27188 29684992 PMC6283711

[7] Lee W-K, Kim M-S, Kang S-W, Kim S, Lee J-R. Type of anaesthesia and patient quality of recovery: a randomized trial comparing propofol-remifentanil total i.v. anaesthesia with desflurane anaesthesia. Br J Anaesth. 2015;114(4):663–8. doi: 10.1093/bja/aeu405 25500679

[8] Kim SH, Ju HM, Choi C-H, Park HR, Shin S. Inhalational versus intravenous maintenance of anesthesia for quality of recovery in patients undergoing corrective lower limb osteotomy: A randomized controlled trial. PLoS One. 2021;16(2):e0247089. doi: 10.1371/journal.pone.0247089 33606764 PMC7894931

[9] Tang L, Sun Y, Hao X, Sun X, Xie C, Wang T, et al. Effect of general anaesthesia with remimazolam versus propofol on postoperative quality of recovery in patients undergoing ambulatory arthroscopic meniscus repair: a randomised clinical trial. BJA Open. 2023;8:100237. doi: 10.1016/j.bjao.2023.100237 37942055 PMC10630608

[10] Kim E-J, Kim C-H, Yoon J-Y, Byeon G-J, Kim HY, Choi E-J. Comparison of postoperative nausea and vomiting between Remimazolam and Propofol in Patients undergoing oral and maxillofacial surgery: a prospective Randomized Controlled Trial. BMC Anesthesiol. 2023;23(1):132. doi: 10.1186/s12871-023-02091-3 37085760 PMC10122311

[11] Zhao SY, Lu XH, Lyu SG, Shan YY, Miao CH. Effects of remimazolam versus propofol on postoperative recovery quality in elderly patients undergoing thoracoscopic laparoscopic radical esophagectomy. Zhonghua Yi Xue Za Zhi. 2023;103(17):1303–9. doi: 10.3760/cma.j.cn112137-20221121-02449 37150679

[12] Luo W, Sun M, Wan J, Zhang Z, Huang J, Zhang J, et al. Efficacy and safety of remimazolam tosilate versus propofol in patients undergoing day surgery: a prospective randomized controlled trial. BMC Anesthesiol. 2023;23(1):182. doi: 10.1186/s12871-023-02092-2 37237331 PMC10214677

[13] Song SW, Kim S, Park J-H, Cho YH, Jeon Y-G. Post-induction hypotension with remimazolam versus propofol in patients routinely administered angiotensin axis blockades: a randomized control trial. BMC Anesthesiol. 2023;23(1):219. doi: 10.1186/s12871-023-02188-9 37349690 PMC10286332

[14] Shimizu T, Takasusuki T, Yamaguchi S. Remimazolam Compared to Propofol for Total Intravenous Anesthesia with Remifentanil on the Recovery of Psychomotor Function: A Randomized Controlled Trial. Adv Ther. 2023;40(10):4395–404. doi: 10.1007/s12325-023-02615-w 37490257 PMC10499674

[15] Zhang L, Wang Z, Liu Y, Zhang X, Wu Y. Comparison of Remimazolam Tosilate and Propofol Sedation on the Early Postoperative Quality of Recovery in Patients Undergoing Day Surgery: A Prospective Randomized Controlled Trial. Drug Des Devel Ther. 2024;18:1743–54. doi: 10.2147/DDDT.S456675 38803562 PMC11129756

[16] Lee J, Han DW, Song Y, Lee J, Jeon S, Kim MH. Quality of Postoperative Recovery in Total Intravenous Anesthesia between Remimazolam and Propofol for Intraoperative Neurophysiological Monitoring: A Prospective Double-Blind Randomized Controlled Trial. J Pers Med. 2024;14(4):382. doi: 10.3390/jpm14040382 38673009 PMC11051443

[17] Choi JY, Lee HS, Kim JY, Han DW, Yang JY, Kim MJ, et al. Comparison of remimazolam-based and propofol-based total intravenous anesthesia on postoperative quality of recovery: A randomized non-inferiority trial. J Clin Anesth. 2022;82:110955. doi: 10.1016/j.jclinane.2022.110955 36029704

[18] Brophy RH, Gefen AM, Matava MJ, Wright RW, Smith MV. Understanding of Meniscus Injury and Expectations of Meniscus Surgery in Patients Presenting for Orthopaedic Care. Arthroscopy. 2015;31(12):2295-300.e5. doi: 10.1016/j.arthro.2015.05.003 26163308

[19] St-Pierre DM. Rehabilitation following arthroscopic meniscectomy. Sports Med. 1995;20(5):338–47. doi: 10.2165/00007256-199520050-00005 8571007

[20] Coetzee JF, Glen JB, Wium CA, Boshoff L. Pharmacokinetic model selection for target controlled infusions of propofol. Assessment of three parameter sets. Anesthesiology. 1995;82(6):1328–45. doi: 10.1097/00000542-199506000-00003 7793646

[21] Minto CF, Schnider TW, Shafer SL. Pharmacokinetics and pharmacodynamics of remifentanil. II. Model application. Anesthesiology. 1997;86(1):24–33. doi: 10.1097/00000542-199701000-00005 9009936

[22] Myles PS, Myles DB, Galagher W, Chew C, MacDonald N, Dennis A. Minimal Clinically Important Difference for Three Quality of Recovery Scales. Anesthesiology. 2016;125(1):39–45. doi: 10.1097/ALN.0000000000001158 27159009

[23] Kampman JM, Hermanides J, Hollmann MW, Gilhuis CN, Bloem WA, Schraag S, et al. Mortality and morbidity after total intravenous anaesthesia versus inhalational anaesthesia: a systematic review and meta-analysis. EClinicalMedicine. 2024;72:102636. doi: 10.1016/j.eclinm.2024.102636 38774674 PMC11106536

[24] Ramirez MF, Gan TJ. Total intravenous anesthesia versus inhalation anesthesia: how do outcomes compare?. Curr Opin Anaesthesiol. 2023;36:399–406. doi: 10.1097/ACO.0000000000001274 37338939

[25] Hung K-C, Wang W-T, Liu W-C, Hsu C-W, Huang Y-T, Wu J-Y, et al. Comparing subjective quality of recovery between remimazolam- and propofol-based total intravenous anesthesia for surgical procedures: a meta-analysis. Syst Rev. 2024;13(1):235. doi: 10.1186/s13643-024-02660-8 39289773 PMC11409698

[26] Song J-L, Ye Y, Hou P, Li Q, Lu B, Chen G-Y. Remimazolam vs. propofol for induction and maintenance of general anesthesia: A systematic review and meta-analysis of emergence agitation risk in surgical populations. J Clin Anesth. 2025;103:111815. doi: 10.1016/j.jclinane.2025.111815 40120546

[27] Wegner BM, Wegner GM, Spagnol LW, Costa LA, Spagnol VW, Paiva DF. Comparison between hemodynamic effects of remimazolam and propofol during general anesthesia: a systematic review and meta-analysis. Minerva Anestesiol. 2024;90(10):901–11. doi: 10.23736/S0375-9393.24.18041-8 39101304

[28] Sessler DI, Bloomstone JA, Aronson S, Berry C, Gan TJ, Kellum JA, et al. Perioperative Quality Initiative consensus statement on intraoperative blood pressure, risk and outcomes for elective surgery. Br J Anaesth. 2019;122(5):563–74. doi: 10.1016/j.bja.2019.01.013 30916004

[29] Kops MS, Pesic M, Petersen K-U, Schmalix WA, Stöhr T. Impact of concurrent remifentanil on the sedative effects of remimazolam, midazolam and propofol in cynomolgus monkeys. Eur J Pharmacol. 2021;890:173639. doi: 10.1016/j.ejphar.2020.173639 33065095

[30] Jonsson Fagerlund M, Krupp J, Dabrowski MA. Propofol and AZD3043 Inhibit Adult Muscle and Neuronal Nicotinic Acetylcholine Receptors Expressed in Xenopus Oocytes. Pharmaceuticals (Basel). 2016;9(1):8. doi: 10.3390/ph9010008 26861354 PMC4812372

[31] Griffin CE 3rd, Kaye AM, Bueno FR, Kaye AD. Benzodiazepine pharmacology and central nervous system-mediated effects. Ochsner J. 2013;13(2):214–23. 23789008 PMC3684331

[32] Ko C-C, Hung K-C, Illias AM, Chiu C-C, Yu C-H, Lin C-M, et al. The use of remimazolam versus propofol for induction and maintenance of general anesthesia: A systematic review and meta-analysis. Front Pharmacol. 2023;14:1101728. doi: 10.3389/fphar.2023.1101728 36814492 PMC9939642

[33] Yao Y, Guan J, Liu L, Fu B, Chen L, Zheng X. Discharge readiness after remimazolam versus propofol for colonoscopy: A randomised, double-blind trial. Eur J Anaesthesiol. 2022;39(12):911–7. doi: 10.1097/EJA.0000000000001715 35796575

[34] Cho E, Roh YH, Moon J, Kim Y, Shin S. Effective bolus dose of remimazolam for i-gel® insertion in nonparalyzed patients: a dose-finding study. Can J Anaesth. 2024;71(9):1251–60. doi: 10.1007/s12630-024-02762-w 38671251 PMC11408568

[35] Chae D, Kim H-C, Song Y, Choi YS, Han DW. Pharmacodynamic analysis of intravenous bolus remimazolam for loss of consciousness in patients undergoing general anaesthesia: a randomised, prospective, double-blind study. Br J Anaesth. 2022;129(1):49–57. doi: 10.1016/j.bja.2022.02.040 35562226

[36] Hu B, Jiang K, Shi W, Xiao S, Zhang S, Zhang Y, et al. Effect of Remimazolam Tosilate on Respiratory Depression in Elderly Patients Undergoing Gastroscopy: A Multicentered, Prospective, and Randomized Study. Drug Des Devel Ther. 2022;16:4151–9. doi: 10.2147/DDDT.S391147 36506792 PMC9733689

[37] Kim H-J, Kim YJ, Lee J, Jeong D, Shin YH, Ro Y-J, et al. Comparison of the recovery profiles of propofol, dexmedetomidine, and remimazolam for intraoperative sedation in patients undergoing upper limb surgery under brachial plexus blockade: a randomized controlled trial. Can J Anaesth. 2025;72(7):1090–100. doi: 10.1007/s12630-025-02987-3 40514628

[38] Wu Q, Xu F, Wang J, Jiang M. Comparison of Remimazolam-Flumazenil versus Propofol for Recovery from General Anesthesia: A Systematic Review and Meta-Analysis. J Clin Med. 2023;12(23):7316. doi: 10.3390/jcm12237316 38068368 PMC10707179

