# Supplementary material for: Quality of recovery after arthroscopic meniscectomy with remimazolam or propofol anesthesia: A randomized controlled trial
Source: PLoS One. 2026 Oct 15;21(10):e0360759. doi: 10.1371/journal.pone.0360759 (PMC13671552; doi:10.1371/journal.pone.0360759)
Supplement: Supplementary file 1 [file pone.0360759.s002.docx]

**S1 Table. Baseline and perioperative characteristics of completers and non-completers**

| **Variable** | **Completers**  **n=71** | **Non-completers**  **n=9** | **P-value** |
| --- | --- | --- | --- |
| Sex, male, n (%) | 34 (47.9) | 5 (55.6) | 0.734 |
| ASA physical status, n (%) |  |  | 0.018* |
| I | 39 (54.9) | 8 (88.9) |  |
| II | 24 (33.8) | 0 (0.0) |  |
| III | 8 (11.2) | 1 (11.1) |  |
| Hypertension, n (%) | 16 (22.5) | 1 (11.1) | 0.676 |
| Diabetes mellitus, n (%) | 5 (7.0) | 1 (11.1) | 0.523 |
| Asthma, n (%) | 4 (5.6) | 0 (0.0) | >0.999 |
| Age, years | 50.5 ± 13.9 | 51.7 ± 12.5 | 0.806 |
| Height, cm | 167.2 ± 10.2 | 164.2 ± 10.6 | 0.411 |
| Weight, kg | 70.9 ± 16.4 | 70.9 ± 16.0 | >0.999 |
| Body mass index, kg·m^-2^ | 25.1 ± 3.9 | 26.1 ± 4.0 | 0.506 |
| Ephedrine requirement, n (%) | 22 (31.0) | 3 (33.3) | >0.999 |
| Ephedrine dose, mg† | 0.0 (0.0-4.0) | 0.0 (0.0-4.0) | 0.853 |
| Remifentanil consumption, µg·kg^-1^·min^-1^ |  |  |  |
| Overall infusion rate † | 0.10 (0.08-0.11) | 0.09 (0.08-0.11) | 0.681 |
| Maintenance infusion rate† | 0.19 (0.15-0.22) | 0.13 (0.10-0.15) | 0.007* |
| Operation time, min† | 30.0 (26.0-36.0) | 30.0 (27.0-30.0) | 0.853 |
| Anesthesia time, min† | 60.0 (55.0-70.0) | 55.0 (50.0-65.0) | 0.479 |
| Time to SGA removal, min† | 11.0 (10.0-14.0) | 10.0 (8.0-11.0) | 0.045* |
| Time to first communication, min† | 14.0 (12.0-16.0) | 12.0 (10.0-13.0) | 0.053 |
| Time to fully alert status, min† | 28.0 (18.0-38.0) | 21.0 (16.0-29.0) | 0.144 |
| PACU duration, min† | 33.0 (30.0-45.0) | 30.0 (30.0-39.0) | 0.432 |

Data are presented as mean ± standard deviation, median (interquartile range), or number (%). † denotes non-normally distributed variables.

ASA, American Society of Anesthesiologists; SGA, supraglottic airway; PACU, post anesthesia care unit.

*P < 0.05
