# Supplementary material for: Quality of recovery after arthroscopic meniscectomy with remimazolam or propofol anesthesia: A randomized controlled trial
Source: PLoS One. 2026 Oct 15;21(10):e0360759. doi: 10.1371/journal.pone.0360759 (PMC13671552; doi:10.1371/journal.pone.0360759)
Supplement: Supplementary file 2 [file pone.0360759.s003.docx]

**S2 Table. Baseline−adjusted and multiple imputation sensitivity analyses of QoR−40 scores on postoperative day 1**

| Domain | Complete−case analysis  (n=71) | | Multiple imputation analysis  (n=80) | |
| --- | --- | --- | --- | --- |
|  | Adjusted mean difference (95% CI) | P−value | Adjusted mean difference (95% CI) | P−value |
| Emotional state | −2.3 (−3.9 to −0.7) | 0.006* | −2.4 (−4.0 to −0.7) | 0.004* |
| Physical comfort | −1.8 (−4.1 to 0.5) | 0.120 | −2.1 (−4.4 to 0.2) | 0.078 |
| Psychological support | 0.4 (−1.3 to 2.0) | 0.677 | 0.4 (−1.3 to 2.1) | 0.656 |
| Physical independence | −0.8 (−2.6 to 1.0) | 0.365 | −0.9 (−2.6 to 0.9) | 0.343 |
| Pain | −0.3 (−2.0 to 1.4) | 0.725 | −0.4 (−2.3 to 1.5) | 0.697 |
| Global score | −5.0 (−11.1 to 1.0) | 0.100 | −5.4 (−11.6 to 0.8) | 0.088 |

Differences are calculated as Propofol group minus Remimazolam group.

Both models were adjusted for age, ASA physical status, asthma, body mass index, and the corresponding preoperative QoR−40 score.

CI, confidence interval; QoR−40, Quality of Recovery−40; ASA, American Society of Anesthesiologists.

*P < 0.05
