# Supplementary material for: Quality of recovery after arthroscopic meniscectomy with remimazolam or propofol anesthesia: A randomized controlled trial
Source: PLoS One. 2026 Oct 15;21(10):e0360759. doi: 10.1371/journal.pone.0360759 (PMC13671552; doi:10.1371/journal.pone.0360759)
Supplement: Supplementary file 3 [file pone.0360759.s004.docx]

**S3 Table. Non-parametric analysis of QoR-40 questionnaire scores**

|  | Propofol group  n=36 | Remimazolam group  n=35 | Hodges-Lehmann location shift (95% CI) | P-value |
| --- | --- | --- | --- | --- |
| Preoperative baseline |  |  |  |  |
| Emotional state | 42.0 (38.0-43.5) | 42.0 (37.0-43.0) | 0.0 (−1.0 to 2.0) | 0.664 |
| Physical comfort | 58.0 (56.0-60.0) | 58.0 (55.0-59.0) | 0.0 (−1.0 to 1.0) | 0.563 |
| Psychological support | 34.0 (31.0-35.0) | 34.0 (29.0-35.0) | 0.0 (−1.0 to 1.0) | 0.971 |
| Physical independence | 25.0 (23.0-25.0) | 25.0 (23.0-25.0) | 0.0 (0.0 to 0.0) | 0.881 |
| Pain | 33.0 (30.0-35.0) | 33.0 (31.0-35.0) | 0.0 (−1.0 to 1.0) | 0.856 |
| Global score | 190.0 (177.0-196.0) | 190.0 (177.0-196.0) | 0.0 (−4.0 to 5.0) | 0.872 |
| Postoperative day 1 |  |  |  |  |
| Emotional state | 39.0 (36.0-39.5) | 39.0 (38.0-41.0) | −1.0 (−3.0 to 0.0) | 0.020* |
| Physical comfort | 54.0 (47.5-55.5) | 55.0 (52.0-56.0) | −1.0 (−3.0 to 1.0) | 0.391 |
| Psychological support | 32.0 (27.0-35.0) | 32.0 (28.0-34.0) | 0.0 (−2.0 to 2.0) | 0.741 |
| Physical independence | 19.0 (15.0-21.0) | 18.0 (15.0-21.0) | 0.0 (−2.0 to 2.0) | 0.699 |
| Pain | 29.0 (26.5-32.0) | 30.0 (27.0-32.0) | 0.0 (−2.0 to 1.0) | 0.685 |
| Global score | 168.0 (156.0-179.5) | 175.0 (163.0-180.0) | −3.0 (−12.0 to 3.0) | 0.306 |

Data are presented as median (interquartile range).

The Hodges-Lehmann location shift is calculated as Propofol group minus Remimazolam group, with P values from the Mann-Whitney U test.

CI, confidence interval; QoR-40, Quality of Recovery-40.

*P < 0.05
