# Supplementary material for: Quality of recovery after arthroscopic meniscectomy with remimazolam or propofol anesthesia: A randomized controlled trial
Source: PLoS One. 2026 Oct 15;21(10):e0360759. doi: 10.1371/journal.pone.0360759 (PMC13671552; doi:10.1371/journal.pone.0360759)
Supplement: Supplementary file 4 [file pone.0360759.s005.docx]

**S4 Table. Perioperative mean arterial pressure and heart rate**

|  | **Unadjusted** | | | **Baseline-adjusted** | | |
| --- | --- | --- | --- | --- | --- | --- |
| Time point | Propofol group  n=36 | Remimazolam group  n=35 | P-value | Propofol group  n=36 | Remimazolam group  n=35 | P-value |
| Mean arterial pressure, mmHg |  |  |  |  |  |  |
| Before induction | 102.8 ± 1.7 | 98.5 ± 1.7 | 0.075 | 103.0 ± 2.3 | 97.9 ± 2.5 | 0.026* |
| After induction | 74.7 ± 2.0 | 74.7 ± 2.1 | 0.994 | 74.9 ± 2.6 | 74.1 ± 2.8 | 0.790 |
| Surgical incision | 75.8 ± 3.0 | 84.5 ± 3.1 | 0.050* | 76.0 ± 3.5 | 83.9 ± 3.6 | 0.075 |
| End of surgery | 79.7 ± 2.0 | 74.4 ± 2.1 | 0.074 | 79.9 ± 2.7 | 73.8 ± 2.8 | 0.042* |
| SGA removal | 81.9 ± 2.2 | 85.5 ± 2.3 | 0.261 | 82.0 ± 2.7 | 84.9 ± 2.9 | 0.353 |
| PACU, 5 min | 91.1 ± 2.6 | 94.0 ± 2.6 | 0.443 | 91.3 ± 3.1 | 93.4 ± 3.3 | 0.577 |
| PACU, discharge | 97.6 ± 2.3 | 96.7 ± 2.3 | 0.786 | 97.8 ± 2.8 | 96.1 ± 3.0 | 0.597 |
| Heart rate, beats·min^-1^ |  |  |  |  |  |  |
| Before induction | 65.8 ± 1.7 | 70.1 ± 1.7 | 0.075 | 64.8 ± 2.4 | 69.0 ± 2.5 | 0.098 |
| After induction | 57.7 ± 1.6 | 66.7 ± 1.6 | <0.001* | 56.7 ± 2.3 | 65.6 ± 2.5 | <0.001* |
| Surgical incision | 56.9 ± 1.4 | 63.5 ± 1.4 | 0.002* | 55.9 ± 2.2 | 62.3 ± 2.4 | 0.003* |
| End of surgery | 57.3 ± 1.5 | 63.7 ± 1.5 | 0.004* | 56.3 ± 2.3 | 62.6 ± 2.5 | 0.008* |
| SGA removal | 63.4 ± 1.9 | 68.5 ± 2.0 | 0.067 | 62.4 ± 2.5 | 67.3 ± 2.7 | 0.076 |
| PACU, 5 min | 60.8 ± 2.0 | 68.1 ± 2.0 | 0.013* | 59.8 ± 2.6 | 66.9 ± 2.7 | 0.015* |
| PACU, discharge | 56.5 ± 1.5 | 66.5 ± 1.5 | <0.001* | 55.5 ± 2.3 | 65.4 ± 2.4 | <0.001* |

Data are presented as estimated marginal mean ± standard error from linear mixed-effects models.

The baseline-adjusted models were additionally adjusted for the baseline characteristics that were imbalanced between groups (standardized mean difference > 0.20): age, ASA physical status, asthma, and body mass index.

SGA, supraglottic airway; PACU, post anesthesia care unit; ASA, American Society of Anesthesiologists.

*P < 0.05
